# Structural and functional insights into metal coordination and substrate recognition of *Akkermansia muciniphila* sialidase Amuc_1547

**DOI:** 10.1186/s43556-025-00265-8

**Published:** 2025-04-23

**Authors:** Tao Li, XinYue Tang, YiBo Zhu, NingLin Zhao, YingJie Song, Lihui He, XingYu Mou, Chunlei Ge, Zhenpu Chen, Hai Zhang, Xiaoxuan Yao, Xiaoyuan Hu, Jiaxing Cheng, Hong Yao, Rui Bao

**Affiliations:** 1 Cancer Biotherapy Center & Cancer Research Institute, Yunnan Cancer Hospital, The Third Affiliated Hospital of Kunming Medical University, Peking University Cancer Hospital Yunnan, Kunming, China; 2 Center of Infectious Diseases, Division of Infectious Diseases in State Key Laboratory of Biotherapy, West China Hospital, Sichuan University, Chengdu, Sichuan 610041 China; 3 Accurate Biotechnology (Hunan) Co, Ltd, Changsha, 410006 China; 4 College of Life Science, Sichuan Normal University, Chengdu, 610101 China; 5 Department of Pharmacy, Chengdu Second People’s Hospital, Chengdu, China

**Keywords:** *A. muciniphila*, Sialidase, Crystal structure, Six-blade β-propeller, CBM-like domain

## Abstract

Sialidases in *Akkermansia muciniphila* are pivotal for mucin degradation, enabling energy acquisition, modulating gut microbiota balance, and influencing host health. However, their structural and functional mechanisms remain poorly characterized. This study resolved the magnesium-bound crystal structure of Amuc_1547, revealing a six-bladed β-propeller fold linked to a carbohydrate-binding module (CBM)-like β-sandwich domain. Structural characterization ​identified a conserved S-x-D-x-G-x-x-W motif, a unique metal-binding pocket coordinated by residues Glu289, Glu299, and Asp300, and a putative carbohydrate substrate-binding pocket within the CBM-like domain. Enzymatic assays confirmed the functional relevance of these structural elements and demonstrated that both metal ions and glycans significantly enhance enzymatic activity. Molecular docking, dynamics simulations, and enzyme kinetics analysis identified critical residue substitutions involved in sialic acid substrate binding and catalysis: Gln367 replaces an arginine in the classical Arg-triplet, while Gln350 and His349 ​replace the nucleophilic tyrosine. These substitutions collectively mediate substrate binding, nucleophilic attack, and transition state stabilization, distinguishing the catalytic mechanism of Amuc_1547 from other six-bladed β-propeller sialidases. Additionally, comparative analysis of the four *A. muciniphila* sialidases highlights sequence divergence and domain architecture variations, suggesting niche-specific roles in gut microenvironments. Our work not only deciphers the structural basis of metal-dependent substrate recognition in Amuc_1547 but also advances our understanding of the adaptation of *A. muciniphila* to gut niches, offering a blueprint for leveraging sialidase-driven mucin metabolism in microbiota-targeted therapies.

**Supplementary Information:**

The online version contains supplementary material available at 10.1186/s43556-025-00265-8.

## Introduction

The sialidases in *A. muciniphila* are important for the bacterium energy metabolism and survival, thereby influencing host health, and are closely correlated with numerous human diseases, including obesity, diabetes, and cardiovascular diseases [1, 2]. These sialidases cleave the sialic acid-containing glycoprotective caps from mucin conjugates, such as N-acetylneuraminic acid (Neu5 Ac) and N-glycolylneuraminic acid (Neu5Gc) [3], which is essential for accessing the underlying sugars and peptide backbones of mucins for further degradation [4]. The products of this degradation not only provide energy for *A. muciniphila* but also allow the gut symbiotic bacteria to metabolize these glycoproteins, thereby promoting the homeostasis of the gut microbiota and enhancing its metabolic capabilities [5, 6].

Sialidases, classified predominantly within the GH33 glycoside hydrolase family [7], catalyze the cleavage of terminal sialic acids from glycoproteins and glycolipids. This activity is central to microbial mucin utilization, enabling nutrient acquisition and shaping host–pathogen interactions [8]. Beyond their metabolic role, sialidases regulate broader biological processes: they mediate microbial adhesion to host surfaces, modulate immune responses, and influence protein aggregation [9]. Critically, dysregulation of sialidase activity contributes to multiple human diseases. For example, in inflammatory bowel disease, altered sialylation disrupts gut microbial-immune crosstalk, exacerbating inflammation [10]. In cancer, sialidase-driven remodeling of cell surface glycans promotes tumor immune evasion and metastasis [11]. Similarly, in Alzheimer’s disease, aberrant neuronal sialylation may accelerate toxic protein aggregation, linking sialidase activity to neurodegeneration [12]. These disease connections underscore the critical need to elucidate sialidase mechanisms, as understanding their roles could inform novel therapies targeting sialidase activity or its downstream pathways.

*A. muciniphila* encodes four sialidases (Amuc_0623, Amuc_0625, Amuc_1835, and Amuc_1547) [13]. which exhibit low sequence homology, suggesting divergent structural and functional adaptations. Among these, Amuc_1547 stands out as a mechanistically unique enzyme. Recent structural studies reveal that Amuc_1547, when complexed with substrate inhibitors and sialyl-T antigen disaccharides, defines a phylogenetically distinct cluster within the GH33 family [4]. Unlike typical GH33 sialidases, Amuc_1547 selectively hydrolyzes sialyl-T antigens in O-glycans, even in the presence of α2,6-sialylation or β1,6-substitutions on GalNAc, a specificity that led to its classification as the founding member of the GH181 family in the CAZy database [4]. Nevertheless, since only one structure for this family has been resolved to date, the precise mechanisms of substrate binding and catalysis by Amuc_1547 remain unclear, necessitating more extensive research to elucidate its functional and mechanistic roles.

This work determined the crystal structure of sialidase Amuc_1547 bound to magnesium ions, revealing a unique and conserved metal ion binding pocket and a potential carbohydrate-binding pocket in the CBM-like domain. Furthermore, functional studies demonstrate that both metal ions and glycans enhance enzymatic activity. Notably, Amuc_1547 employs a non-canonical catalytic triad (Gln367, Gln350, and His349) diverging from the classical arginine triplet and nucleophilic Tyr/Glu pairs of GH33 sialidases. Subsequent molecular docking predictions revealed key residues in Amuc_1547 involved in substrate binding with SIA and 6'-Sialyllactose (6'SL), and mutagenesis experiments confirmed that mutations at these key residue sites reduced Amuc_1547 activity, while mutagenesis of key residues in the catalytic and substrate-binding sites significantly reduces hydrolysis. These findings advance mechanistic insights into GH181 sialidases and provide a foundation for exploring their roles in host-microbe interactions and disease.

## Results

### Overall structure of Amuc_1547

**Fig. 1:**
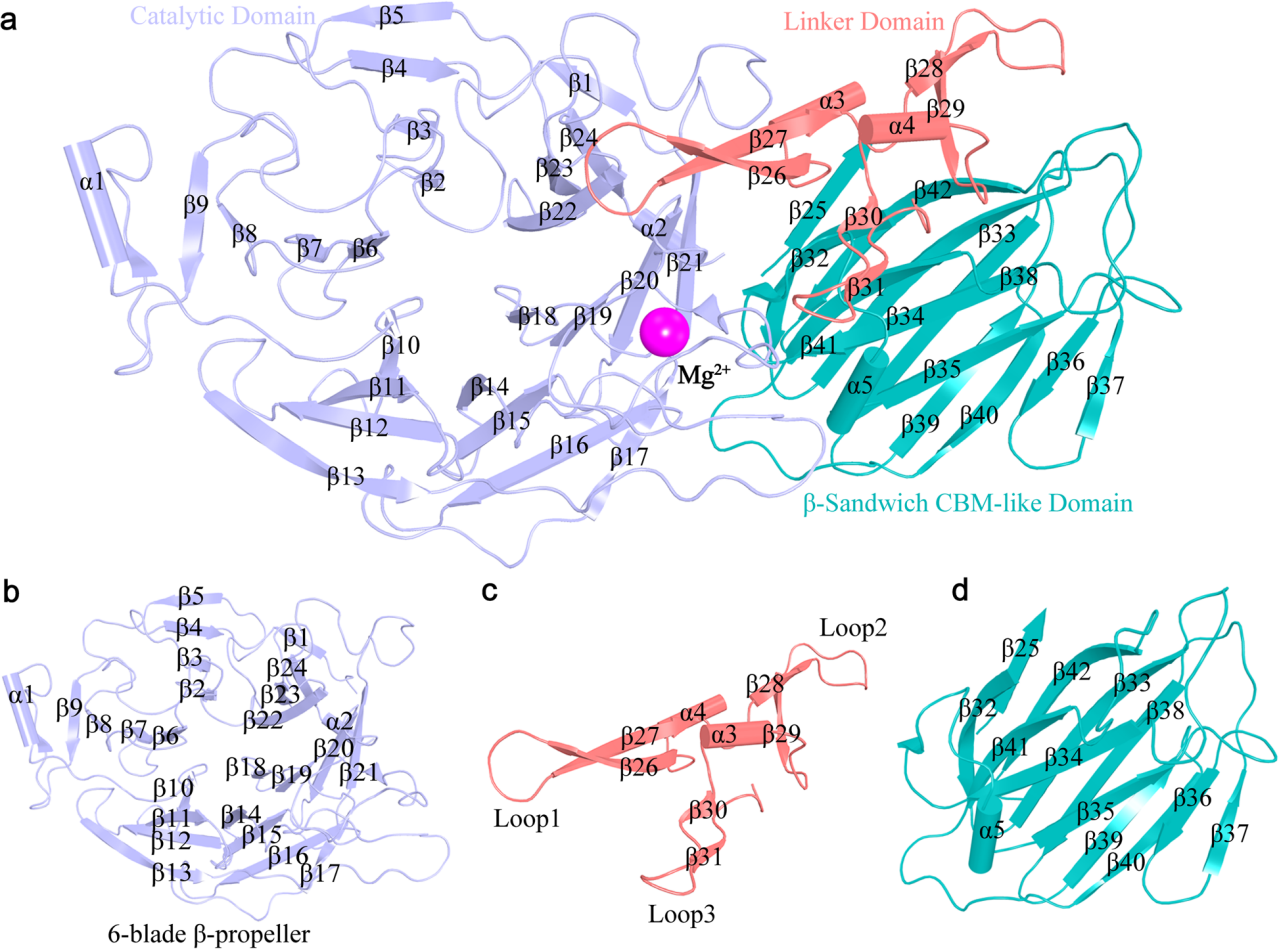
Overall Structure of Amuc_1547. **a** The secondary structure of Amuc_1547 is depicted using both cartoon and surface rendering. **b** The catalytic domain at the N-terminus (residues 19–381) in Amuc_1547. **c** The central linker domain (residues 382–456). **d** The carbohydrate-binding module-like (CBM-like) domain at the C-terminus (residues 457–595)

The crystal structure of Amuc_1547 was solved via single wavelength anomalous dispersion (SAD) using selenomethionine labeled Amuc_1547 (Supplementary Fig. 1). The structure was optimized to a high resolution of 2.0 Å, with final R_work_ and R_free_ values of 17.97% and 22.83%, respectively (Supplementary Table 1). Amuc_1547 is composed of three distinct structural domains: the catalytic domain at the N-terminus (residues 19–381), the central linker domain (residues 391–456), and the carbohydrate-binding module-like (CBM-like) domain at the C-terminus (residues 457–595) (Fig. 1a).

The catalytic domain is composed of six blades, and each blade is typically composed of around antiparallel four β-sheet. All blades are arranged in a counterclockwise direction around the central axis of the catalytic pocket in a propeller-like structure (six-blade β-propeller). This central catalytic region plays an essential role in both molecular recognition processes and the subsequent hydrolytic cleavage of substrates through precise binding interactions (Fig. 1b).

The linker domain is composed of two alpha-helices and six beta-strands, with four beta-strands forming loops 1 and 3 in a pairwise and antiparallel pattern, and the other two beta-strands forming loop 2. These loops interact with the catalytic domain and the CBM-like domain. The two alpha-helices are located on the periphery of the beta-strands, providing stability to the overall conformation of the linker domain (Fig. 1c).

The CBM-like domain consists of twelve antiparallel beta-strands and a single alpha-helix, with six beta-strands forming an approximately parallel interface, collectively forming two β sheets. The alpha-helix located on the side near the catalytic domain of the two layers, forming a unique β-sandwich fold conformation (Fig. 1d).

### A unique metal ion-binding pocket and the canonical S-x-D-x-G-x-x-W motif across β-propeller blades discovered in Amuc_1547

**Fig. 2:**
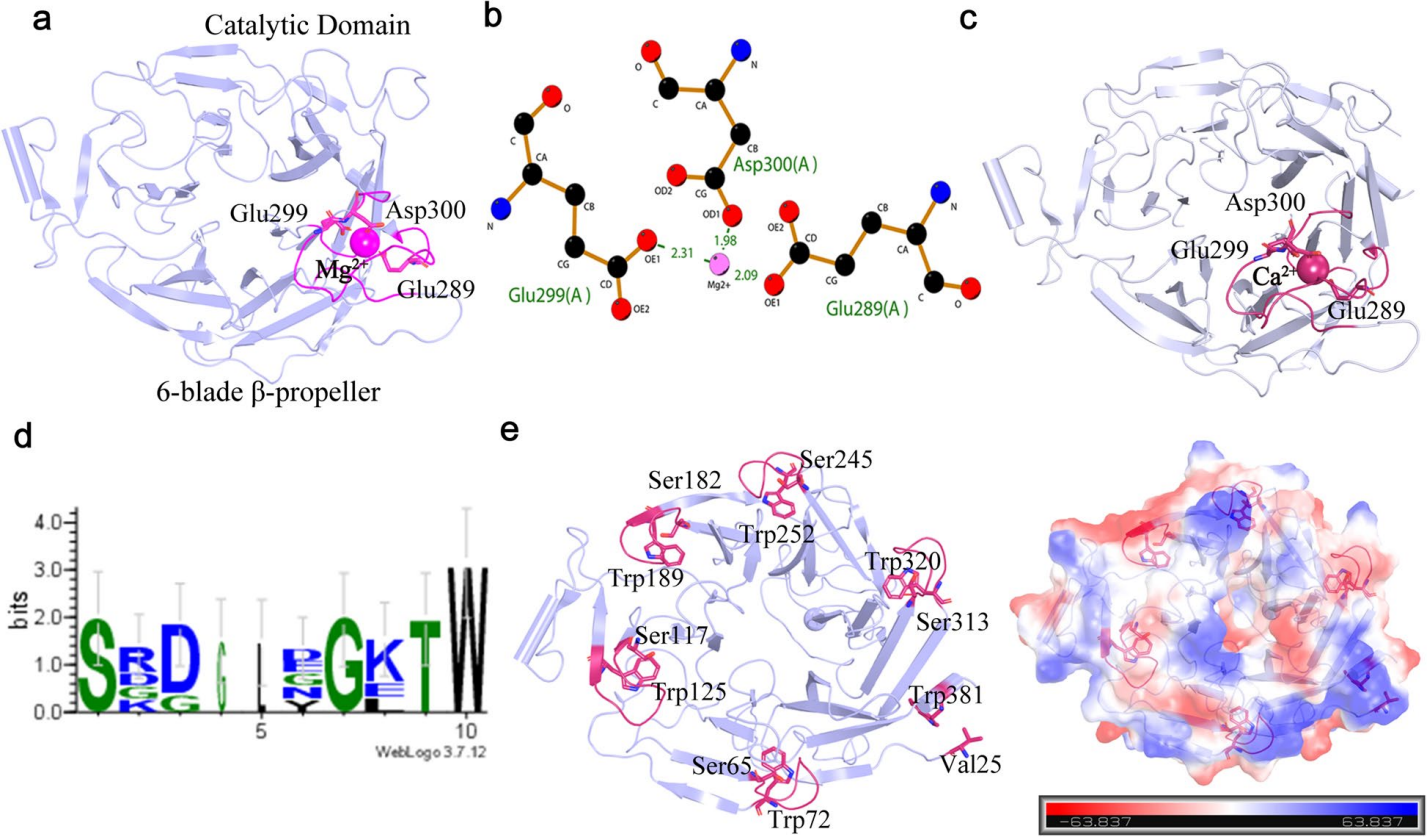
Structural Features of the N-Terminal Catalytic Domain of Amuc_1547. **a** Magnesium Ion Binding Pocket in Amuc_1547 (PDB ID: 8HLS): The magnesium ion binding pocket is highlighted, featuring key loop regions (residues 285–304, 209–213) displayed in hot pink. Coordinating amino acids Glu289, Glu299, and Asp300 are presented in stick model. **b** The interactions of amino acids within the magnesium ion (Mg^2+^) binding pocket are depicted using cLigplus software. **c** Calcium ion (Ca^2+^) binding pocket in Amuc_1547 (PDB ID: 8 AXI). **d** Conservation of the S-x-D-x-G-x-x-W Motif in Amuc_1547 generated by WebLogo (https://weblogo.berkeley.edu/logo.cgi). **e** Location of the S-x-D-x-G-x-x-W Motif in the Catalytic Domain. This motif is located within the six-bladed beta-propeller of the catalytic domain. Residues S and W are displayed in stick representation, with the charge distribution generated by PyMOL. Red indicates negatively charged regions, while blue signifies positively charged areas

Further structural analysis revealed that the catalytic domain of Amuc_1547 contains a magnesium (Mg^2^⁺) ion-binding pocket (Fig. 2a), coordinated by three amino acids: Glu289, Glu299, and Asp300 (Fig. 2b). In previously reported structures [4], this same region is coordinated with calcium ions (Ca^2^⁺) via the same amino acids, suggesting that this is a conserved metal ion-binding pocket in Amuc_1547 (Fig. 2c).

Subsequently, We aligned Amuc_1547 with representative GH33 family members, which have a six-blade β-propeller fold and high sequence homology [14]. Results showed Glu289, Glu299, and Asp300 in the metal ion-binding pocket aren't conserved in GH33 (Supplementary Fig. 2). We also predicted the structures of three other *A. muciniphila* sialidases (Amuc_0623, Amuc_0625, Amuc_1835), whose catalytic domains all share with six-blade β-propeller folding pattern (Supplementary Fig. 3). Sequence alignment revealed these three sialidases also don't conserve the three amino acids (Supplementary Fig. 4), indicating this metal ion-binding pocket is unique to Amuc_1547.

Further sequence alignment with members of the GH33 family revealed the presence of six conserved S-x-D-x-G-x-x-W motifs within the six-blade β-propeller catalytic domain (Fig. 2d, Supplementary Fig. 2). These motifs are located on the β-hairpin loops formed between the third and fourth β strands, positioned on the outer side of each blade of the six-blade propeller structure (Fig. 2e). The serine (S) and tryptophan (W) residues in these motifs anchor the ends of the β-hairpin loops, while the -x-D-x-G-x-x- sequence engages in charge interactions with amino acids on neighboring β-propeller blades (Fig. 2e), thus ensuring the counterclockwise orientation of the propeller blades and the structural folding stability. It is also significant to note that these motifs exhibit partial conservation in other three sialidases from *A. muciniphila* (Amuc_0623, Amuc_0625, Amuc_1835) (Supplementary Fig. 4).

These results indicate that Amuc_1547 possesses a unique and conserved metal ion binding pocket coordinated by Glu289, Glu299, and Asp300, as well as a widely conserved S-x-D-x-G-x-x-W motif, which maintains the folding conformation of the six-blade β-propeller.

### The interaction interface of the linker domain supports the structural stability of Amuc_1547

**Fig. 3:**
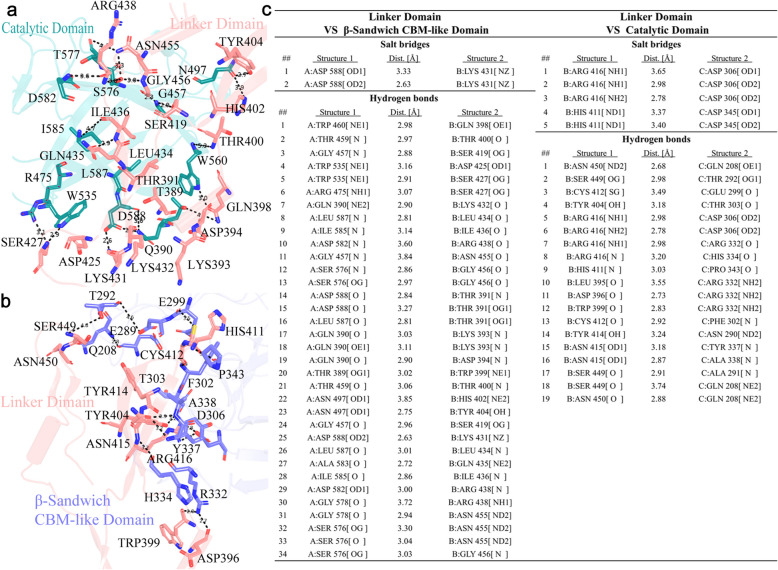
Interactions between the linker domain and catalytic, as well as CBM-like domains in Amuc_1547. **a** The amino acid residues involved in the interaction between the linker domain and the catalytic domain are displayed in a stick model, with interacting residues in the linker domain shown as light gray sticks and those in the catalytic domain shown as pink sticks. **b** The amino acid residues involved in the interaction between the linker domain and the CBM-like domain are displayed in a stick model, with interacting residues in the CBM-like domain shown as cyan sticks and those in the catalytic domain shown as pink sticks. **c** Detailed information on the key residue interactions at the interface, including salt bridges, hydrogen bonds, and the distances between them

The interaction interface of the linker domain maintains the stability of the overall conformation of Amuc_1547. The interaction interface areas between the linker domain and the catalytic domain, as well as the CBM-like domain, are 1047.3 Å^2^ and 1217.0 Å^2^, respectively. These interaction interfaces are stabilized through salt bridges, hydrogen bonds, and hydrophobic interactions (Supplementary Fig. 5). Specifically, the stability between the linker domain and the catalytic domain is maintained by 2 salt bridges and 19 hydrogen bond interactions, while the stability between the linker domain and the CBM-like domain is maintained by 1 salt bridge and 34 hydrogen bond interactions (Fig. 3 a, b & c). These interactions ensure the key role of the linker domain in maintaining the stability of the overall conformation of Amuc_1547.

### The CBM-like domain of Amuc_1547 harbors a potential binding pocket for carbohydrate substrates

**Fig. 4:**
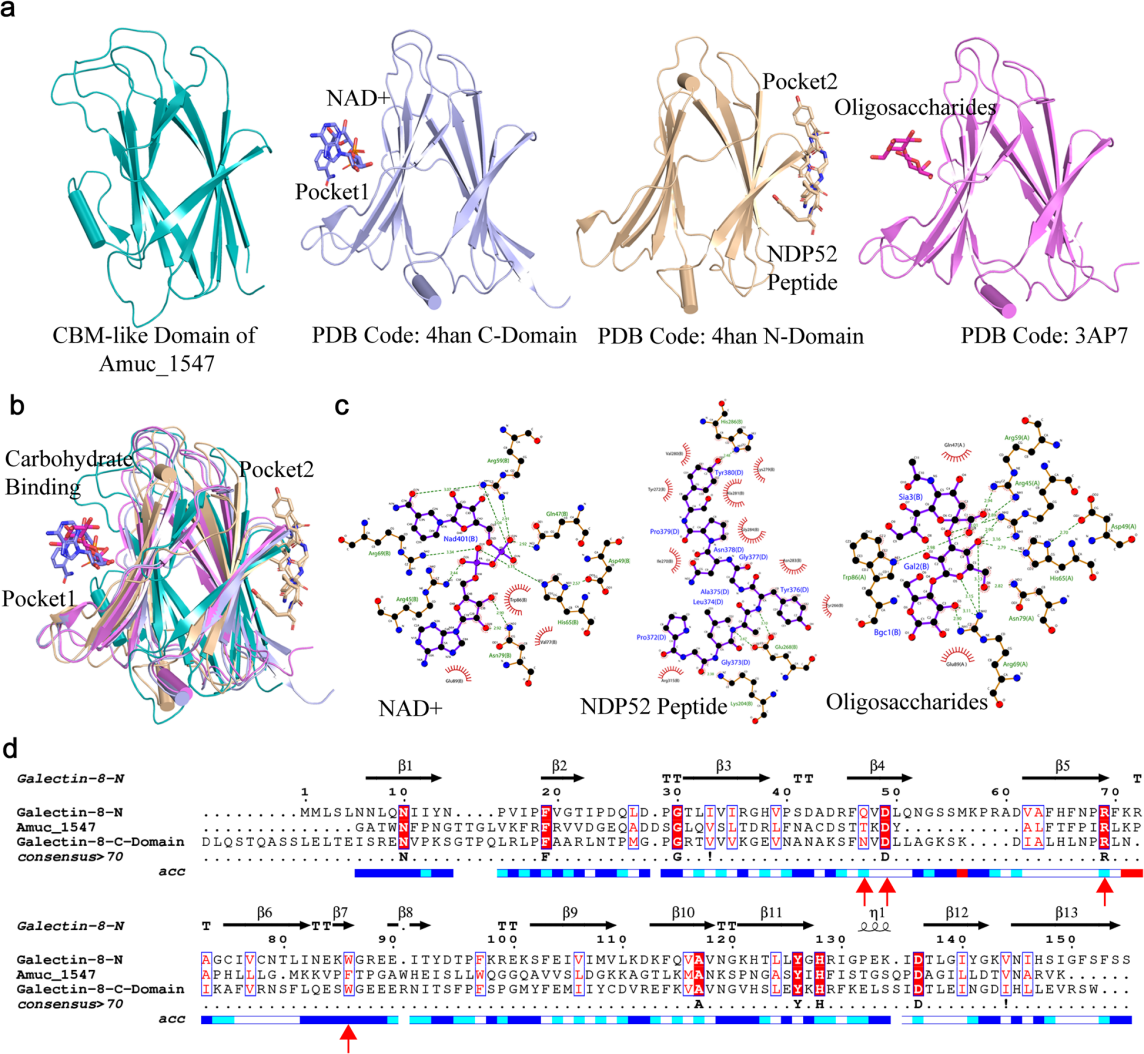
Analysis of the CBM-like domain of Amuc_1547 and its potential binding pockets. **a** Structural homology and substrate binding sites. The structural homology between Amuc_1547 and two high-scoring structures, 4HAN and 2 AP7, using the DALI server. Both structures are shown with their respective binding pocket for NAD +, oligosaccharides, and NDP52 peptide on the beta-sandwich fold. Pocket1 for NAD + or oligosaccharides, and Pocket2 for NDP52 Peptide. **b** The NAD + and oligosaccharides binding sites are found to be within the same pocket. **c** Key amino acid sites for NAD +, NDP52 peptide and oligosaccharides binding, which generated by cLigplus. **d** Sequence alignment and conservation. Conserved residues binding NAD + and oligosaccharides are marked with red arrows

We used the DALI server to search for homologous structures to the CBM-like domain of Amuc_1547. Significant homology was found with Homo sapiens Galectin- 8, a beta-galactoside-binding lectin [15]. We compared Amuc_1547 with two high-scoring structures (PDB Code: 4HAN, 3 AP7) both of which are Galectin- 8. Notably, in the 4HAN structure [16], Galectin- 8 can be divided into two structural domains that exhibit similar sequences and folding patterns. The N-terminus binds nicotinamide adenine dinucleotide (NAD +), and the C-terminus binds NDP52 peptide, with both binding pockets located on opposite sides of the beta-sandwich fold (Pocket1 for NAD +, Pocket2 for NDP52 Peptide) (Fig. 4a). In the 3 AP7 structure [17], the N-terminus of Galectin- 8 binds oligosaccharides (Fig. 4a), and the comparison shows that NAD + and oligosaccharides are bound within the same pocket (Fig. 4a & b).

Upon analyzing the residues binding NAD + and oligosaccharides in both structures, we found that Arg59, Arg45, Asp49, His65, Arg69, and Asn79 are shared key residues for both substrates, while the key residues for binding NDP52 peptide are His286, Glu268, and Lys204 (Fig. 4b & c). Subsequent sequence alignment revealed that Asp49 and Arg69 are completely conserved with Asp505 and Arg514 in Amuc_1547, respectively (Fig. 4d). This indicates that the CBM-like domain of Amuc_1547 contains a pocket composed of polar amino acids such as arginine, which may potentially bind to NAD +, oligosaccharides, or other carbohydrate substrates.

### The unique catalytic residues (Gln367, His349, and Gln350) in Amuc_1547 set it apart from other sialidases and the GH33 family

**Fig. 5:**
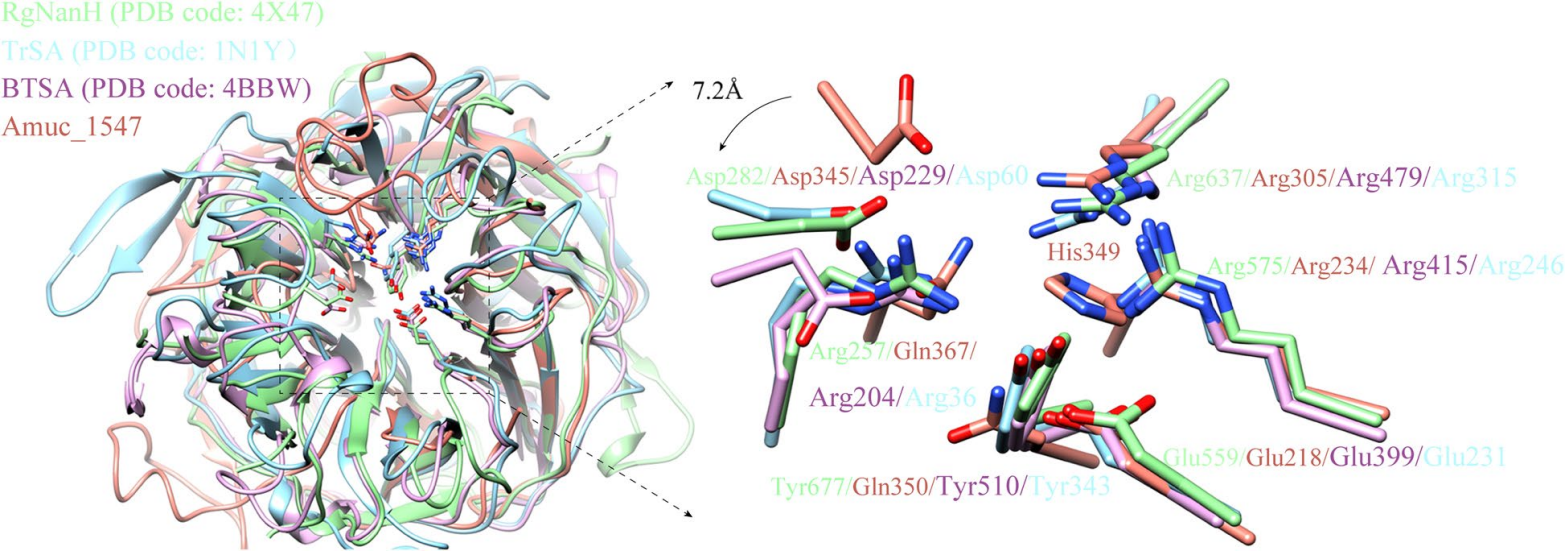
Structural comparison of catalytic centers of Amuc_1547 with other GH33 sialidases. RgNanH (PDB code: 4X47) in light green, TrSA (PDB code: 1 N1Y) in light blue, BTSA (PDB code: 4BBW) in lilac, and Amuc_1547 in rose red. The GH33 family catalytic active center residues, including an arginine triplet (3 Arg), a pair of nucleophilic amino acids (Glu and Tyr), and an acid–base catalytic amino acid (Asp) were displayed in stick

We compared the catalytic active sites of Amuc_1547 with representative structures of the GH33 family (Fig. 5). The characteristic features of the GH33 family catalytic active center residues are three arginines (Arg-triplet), a pair of nucleophilic amino acids (Glu and Tyr), and an acid–base catalytic amino acid (Asp) [18]. This feature has also been reported in other six-blade β-propeller families, such as GH34 and GH83 (Fig. 5). In Amuc_1547, the characteristic residue triplet coordinating with the carboxylate group only includes two arginine (Arg234 and Arg305) and a substituted glutamine (Gln367) (Fig. 5). The nucleophilic residue (Glu218) and an acid–base catalytic amino acid Asp345 are retained, likely playing the role of a general acid–base catalytic residue, consistent with other typical sialidases (Fig. 5). Notably, another conserved nucleophilic residue, tyrosine (Tyr), is replaced in Amuc_1547 by two different residues, His349 and Gln350, both of which possess nucleophilic attack capability.

Interestingly, when we examined the distribution of these conserved amino acids among the four different sialidases of *A. muciniphila,* we found the unique catalytic amino acids Gln367, His349, and Gln350 in Amuc_1547 are not conserved in the other three sialidases (Supplementary Fig. 4), suggesting that the four sialidases in *A. muciniphila* may have different substrate catalytic activities.

### A potential substrate binding pocket in the catalytic domain of Amuc_1547

To better understand the mechanism by which Amuc_1547 catalyzes sialic acid, molecular docking was performed using the apo structure, resulting in docking complex for SIA and 6'SL by Autodock4.2.6. Molecular docking analysis revealed that the lowest-energy cluster dominated the conformational ensemble, with the majority of poses ​predominantly clustered within this region and exhibiting stable binding conformations (Supplementary Fig. 6). Specifically, for SIA, 147 out of 200 docking poses (73.5%) fell into the lowest energy cluster, while for 6'SL, due to its larger molecular weight as a trisaccharide compared to SIA, 47 poses (23.5%) were found in the lowest energy cluster, which was the largest proportion for 6'SL (Supplementary Fig. 6).

**Fig. 6:**
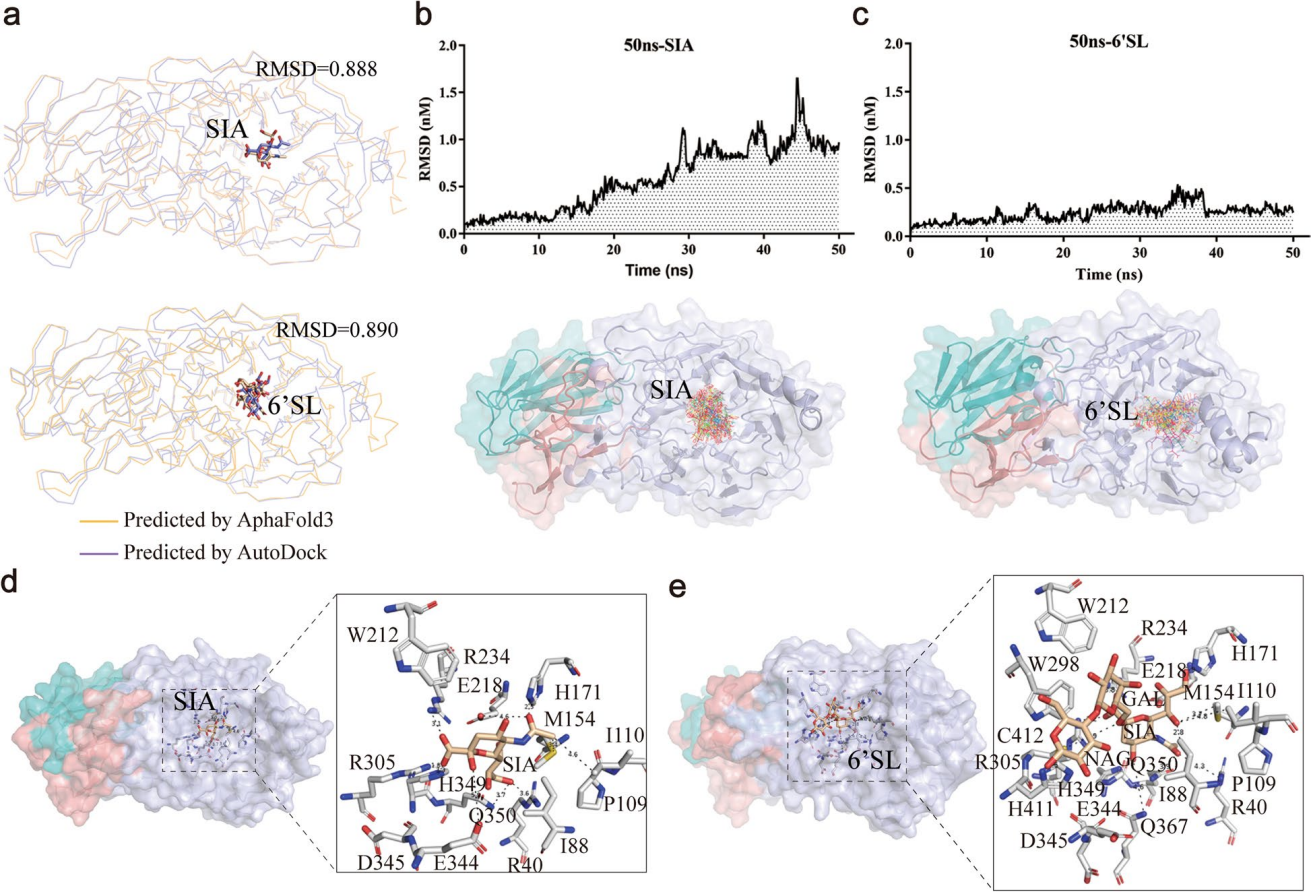
The molecular docking structure of Amuc_1547 binding to sialic acid substrate. **a** Comparison of the AutoDock and AlphaFold3 predicted complexes of Amuc_1547 binding to sialic acid substrate, SIA, and 6'SL. **b-c** 50 ns molecular dynamics simulation results of Amuc_1547 binding to SIA and 6'SL, respectively. The upper panel shows the RMSD statistics, and the lower panel displays the substrate docking results at various time points within the 50 ns simulation. **d-e** Interaction of key residues involved in substrate binding in the AlphaFold3 predicted complex of Amuc_1547 with substrate SIA (**d**) and 6'SL (**e**). 6'SL (6'-Sialyllactose): A glycoside compound composed of Sialic acid (SIA), Galactose (GAL), and N-Acetylglucosamine (NAG)

To further validate the reliability of the docking results, we performed docking calculations for the substrates SIA and 6'SL using AlphaFold3, with IPTM values of 0.65 and 0.45, respectively. The docking results from both AlphaFold3 and AutoDock, showing binding in the same substrate-binding pocket with Cα RMSD values of 0.888 and 0.89 for SIA and 6'SL, respectively (Fig. 6a), further enhance the reliability of the molecular docking results. Additionally, we conducted 50 ns molecular dynamics simulations, and the results showed that during the 50 ns simulation, SIA and 6'SL stably bound to the substrate-binding pocket, with RMSD values remaining below 1.5 nm (Fig. 6b & c). The dual validation from both docking schemes and molecular dynamics simulations fully confirmed the reliability of Amuc_1547 binding to the substrates SIA and 6'SL.

Furthermore, we performed a detailed analysis of the highly recognized AlphaFold3 docking complex structures Amuc_1547-SIA and Amuc_1547 - 6'SL. In both structures, SIA is located at the bottom of the binding pocket, and several key amino acid residues, including Arg234, Arg305, Gln367, Arg40, Trp212, Glu218, His171, Asn214, Gln350, Asp345, Gln367, and His349, interact with the substrate SIA (Fig. 6d & e). The hydrophobic pocket formed by amino acids such as Ile88, Ile110, Pro109, and Met154 accommodates the N-acetyl group of SIA (Fig. 6d & e). In addition, in the Amuc_1547 - 6'SL complex, several distinct substrate recognition features are observed, with His411, Cys412 Gln367, Trp212, and Trp298 are involved potentially contributing to the recognition of galactose (GAL) moiety and N-acetylglucosamine (NAG) (Fig. 6e).

### Key residue mutations in the substrate binding pocket and CBM-like domain deletion mutants both reduce the sialidase activity of Amuc_1547

**Fig. 7:**
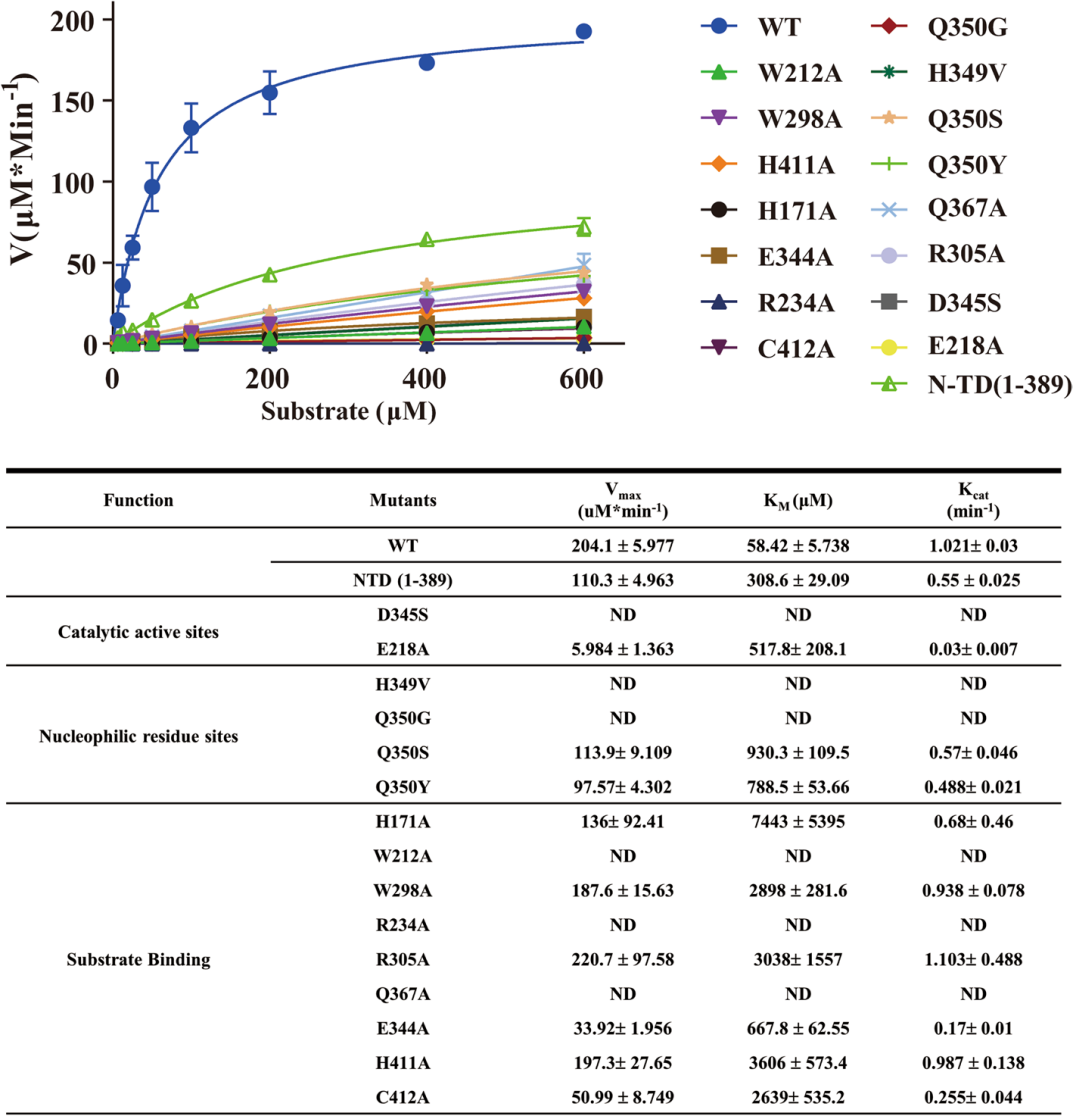
Kinetic analysis and parameters of Amuc_1547 enzymes and mutants. All mutant proteins (purity > 90%) were purified using the same protease method as the wild-type. All enzymes (400 nM) were incubated with varying concentrations of 4MU-Neu5 Ac (6.25 μM- 600 μM) in 25 mM Tris, 150 mM NaCl, pH 7.5 at 37 °C. Apparent activation parameters of Amuc_1547 and mutants were determined using the formula of Michaelis–Menten [Y = Vmax*X/(Km + X)], Prism 8. The parameters listed are the means of three independent determinations. ND, no detectable activity

Furthermore, we established an enzyme activity assay used to measure the activity of sialidase, employing the fluorescent substrate 4MU-Neu5 Ac to determine the substrate catalytic activity of crucial catalytic residues in sialidase [19]. Based on the molecular docking analysis of the key residues mentioned above, we expressed and purified the mutant proteins and measured their activity against the fluorescent substrate 4MU-Neu5 Ac (Fig. 7). The results showed that the wild-type Amuc_1547 had a Vmax of 204.1 ± 5.977 (μM*min⁻^1^), Km of 58.42 ± 5.738 μM, and a Kcat value of 1.021 ± 0.03 min⁻^1^. Further kinetic parameters of the mutants showed that the maximum degradation rates for mutants at substrate binding-related sites (H171 A, W212 A, W298 A, R234 A, R305 A, Q367 A, E344 A, H411 A, C412 A), catalytic activity-related sites (D345S, E218 A), and nucleophilic residue-related sites (H349 V, Q350G) all showed reductions or complete loss of activity, and the Km values increased or could not be measured (Fig. 7, Supplementary Fig. 7& Supplementary Table 2). Notably, compared to the Q350G mutant, which cannot provide nucleophilic attack, the Q350S and Q350Y mutants exhibited some recovery in activity, though still lower than the wild type. This may be due to the inability of the H349 and S350 combination to perform strong nucleophilic attack, as well as the steric hindrance between H349 and Y350, leading to reduced activity. This emphasizes that the H349 and Q350 combination is the optimal alternative to the classic tyrosine for nucleophilic attack in other six-blade β-propeller families. All these data further highlight the importance of the key residue sites in the activity of Amuc_1547.

### Amuc_1547 senses metal ions and carbohydrates (NAD + and oligosaccharides) to enhance its sialidase activity

**Fig. 8:**
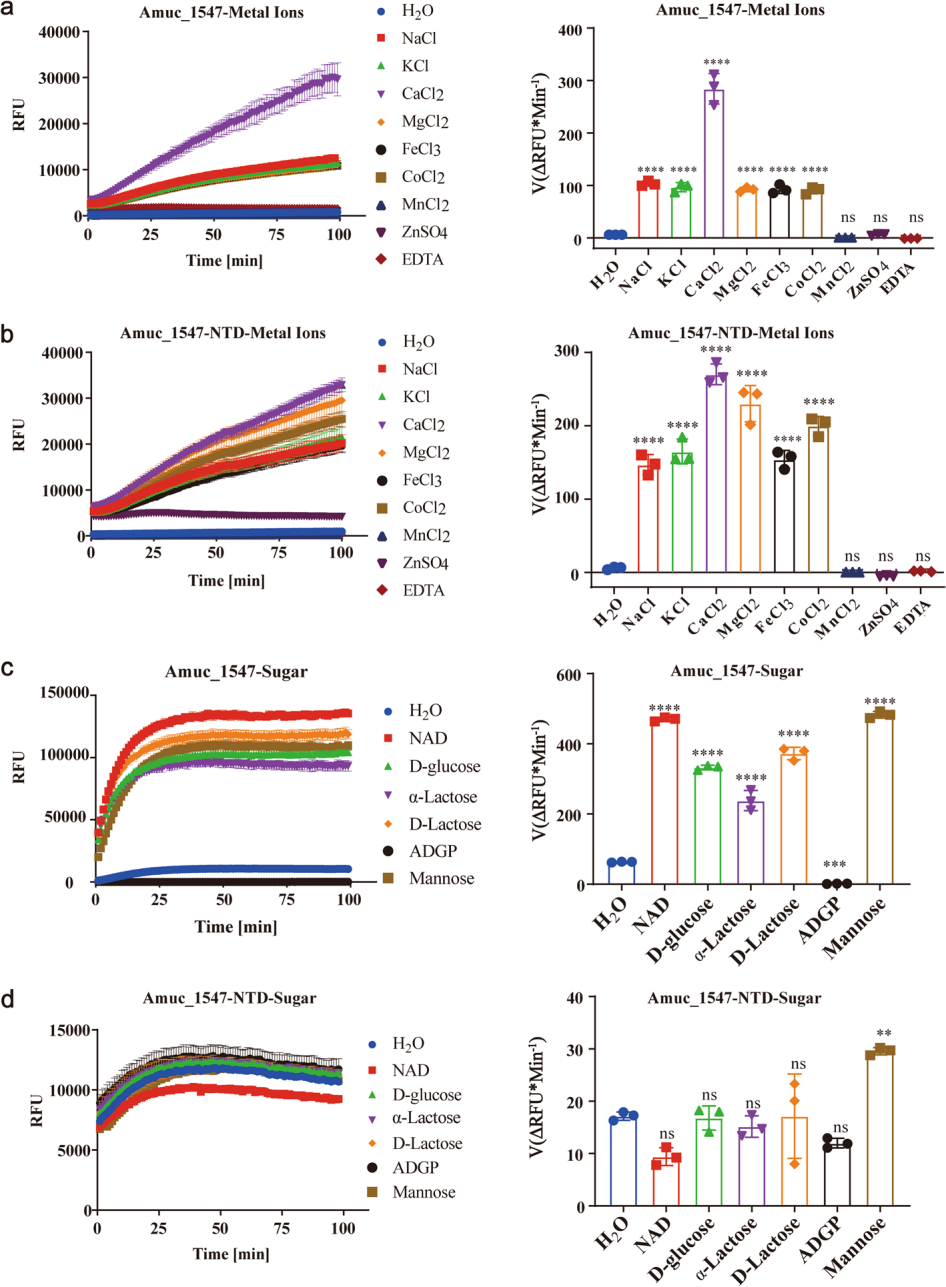
Determination of enzyme activity of Amuc_1547 and Amuc_1547-NTD to ions and carbohydrate (NAD + and oligosaccharides). **a-b** Effect of different metal ions on the activity of Amuc_1547 and Amuc_1547-NTD. **c-d** Effect of NAD + and oligosaccharides on the activity of Amuc_1547 and Amuc_1547-NTD. (Left) Fluorescence change over time, (Right) Statistical analysis of substrate fluorescence generation rates. For ions and carbohydrate (NAD + and oligosaccharides) effect tests, enzymes (400 nM) were incubated with 100 mM 4MU-Neu5 Ac in enzyme-free water at 37 °C. Final concentrations of all ions and carbohydrates were 2 mM. The reaction volume was 100 μL, and progress was monitored via fluorescence (excitation 373 nm, emission 448 nm) using a TECAN Spark® Multi-Mode Microplate Reader. The Kinetic parameters listed are the means of three independent determinations. Error bars represent standard error of the mean (s.e.m.). Note: ADGP is the abbreviation for Alpha-D-glucopyranose pentabenzoate

To further clarify the effects of metal ions and the CBM-like domain binding carbohydrates (NAD + and oligosaccharides) on the catalytic activity of Amuc_1547, we generated three truncated variants based on the domain structure outlined in Fig. 1, including Amuc_1547-NTD (deleting the CBM domain, amino acids 1–381), Amuc_1547-CBM-like domain1 (381–595), and Amuc_1547-CBM-like domain2 (465–595). Unfortunately, we were only able to obtain the Amuc_1547-NTD protein (Supplementary Fig. 7, Supplementary Table 2). Subsequently, we measured the impact of metal ions and carbohydrates (NAD + or oligosaccharides) on the activity of Amuc_1547 and Amuc_1547-NTD in the presence of only enzyme-free water. The results showed that various metal ions, including sodium, magnesium, calcium, iron, and cobalt, significantly enhanced the activity of both Amuc_1547 and Amuc_1547-NTD (Fig. 8a & b). In contrast, zinc and manganese did not significantly affect enzyme activity, indicating a selective interaction with specific metal ions (Fig. 8a & b).

Similarly, we further investigated the effect of carbohydrates, including NAD + and oligosaccharides (D-glucose, α-lactose, D-lactose, Alpha-D-glucopyranose pentabenzoate, Mannose), and found that the addition of NAD + and oligosaccharides (Alpha-D-glucopyranose pentabenzoate was ineffective, possibly due to its poor solubility) significantly increased the enzyme activity of Amuc_1547 (Fig. 8c). In contrast, the activity of Amuc_1547-NTD was almost unaffected by NAD + and oligosaccharides, which may be due to the absence of the CBM-like domain, preventing it from sensing NAD + and oligosaccharides (Fig. 8d). These data demonstrate that Amuc_1547 has selective ion binding, which is crucial for its activity. Furthermore, the CBM-like domain enhances sialidase activity by sensing NAD + or oligosaccharides, thereby improving the survival of *A. muciniphila* in the complex gut environment.

## Discussion

*A. muciniphila* is a symbiotic gut bacterium associated with various human diseases such as obesity, diabetes, and cardiovascular diseases, and it can regulate host health [1]. Sialidases, essential for mucin degradation to support bacterial survival and gut health, remove sialic acid from mucins, enabling nutrient acquisition by *A. muciniphila* and other gut bacteria, with ​Amuc_1547 representing a key sialidase in *A. muciniphila*. In this study, we present its crystal structure under different crystallization conditions, which includes the binding of a magnesium ion. Subsequently, through detailed structural analysis and comparison, we identified a unique and conserved metal ion coordination binding pockets that coordinates by Glu289, Glu299, and Asp300, and the widely conserved S-x-D-x-G-x-x-W (Figs. 1 and 2). Furthermore, a potential carbohydrate-binding pocket is observed (Fig. 4), which suggests an interaction site for carbohydrate substrates. In addition to sialidases catalytic, metal ions are crucial for maintaining the structural integrity of Amuc_1547 and stabilizing the transition state during catalysis. The coordination of metal ions with key residues in the catalytic domain ensures the enzyme maintains its active conformation and functionality. Our data show that various metal ions, including sodium, magnesium, calcium, iron, and cobalt, enhance Amuc_1547 activity, suggesting the enzyme’s ability to adapt to diverse physiological conditions where these ions may be abundant (Fig. 8). In contrast, zinc and manganese did not significantly impact enzyme activity, indicating a selective interaction with specific metal ions. This heterogeneity in sialidase genes and functions may stem from the need for rapid adaptation to the constantly changing gut environment in humans, thereby increasing the redundancy of *A. muciniphila* in regulating host health within the gut.

Carbohydrate-Binding Modules (CBMs) are structural domains widely found in glycoside hydrolases, esterases, and oxidoreductases [20]. Their primary function is to enhance enzyme–substrate affinity and catalytic efficiency by specifically recognizing and binding to polysaccharide substrates, such as cellulose, chitin, and mucins [21]. We identified that the CBM-like domain of Amuc_1547 exhibits a distinct β-sandwich folding pattern (Fig. 1d). Homology analysis revealed that the CBM-like domain of Amuc_1547 shares high structural and sequence homology with human Galectin- 8 (Fig. 2). Further structural characterization identified a conserved carbohydrate-binding pocket within this domain, composed of arginine residues (Fig. 2, Supplementary Fig. 2), suggesting functional parallels with Galectin- 8 in substrate recognition. Furthermore, enzymatic assays demonstrated that NAD⁺ and specific oligosaccharides significantly enhance Amuc_1547 activity (Fig. 8a). Strikingly, truncation of the CBM-like domain (Amuc_1547-NTD) abolished this responsiveness (Fig. 8), confirming the critical role in sensing carbohydrate ligands. These findings emphasize that the CBM-like domain plays a key role in enhancing sialidase activity by sensing NAD + or oligosaccharides, thereby contributing to the survival and adaptability of *A.muciniphila* in the complex and fluctuating environment of the gut microbiota.

The classic sialidase active center (GH33 family) has an arginine triplet, a nucleophilic pair (glutamate and tyrosine), and an acid–base catalytic aspartate [18]. The positive charge of the arginine stabilizes the sialic acid carboxylate group. The nucleophilic tyrosine residue, with its hydroxyl oxygen atom, attacks the carbon atom of the substrate sialic acid, forming a transition state stabilized by glutamate and aspartate. Surprisingly, Amuc_1547 has unusual catalytic residues: Gln367, Gln350, His349, and conserved sites (Arg234, Arg305, Glu218, Asp345) (Fig. 5). An arginine in the triplet is replaced by Gln367, and the nucleophilic tyrosine is replaced by His349 and Gln350, both with nucleophilic ability. Considering that glutamine is more nucleophilic than histidine and given the significant steric hindrance of His349 imidazole ring, we believe that Gln350 is the most likely candidate to act as the nucleophilic residue. It replaces the conserved tyrosine residue and exerts nucleophilic attack through its side chain amino group (Fig. 5&6). However, unlike tyrosine with a large aromatic side chain, the side chain of Gln350 is relatively small and more flexible, which may require the adjacent His349 to help stabilize it. Furthermore, since the histidine in the catalytic center of EnvSia156 has been reported to act as a proton donor to stabilize the transition state [22]. His349 in Amuc_1547 may serve a dual role: stabilizing the catalytic residue Gln350 and providing a proton for the transition state.

Sialidases are glycoside hydrolases that catalyze the cleavage of terminal sialic acid (SIA) residues from glycoconjugates, including mucins and glycolipids [23]. In this study, we focused on two key substrates: free sialic acid (SIA) and 6'-sialyllactose (6'SL), a trisaccharide abundant in human milk and intestinal mucins. Molecular docking results of the N-terminal catalytic domain binding to substrates SIA and 6'SL in Amuc_1547, along with comparisons to other families with a six-bladed propeller folding pattern, reveal distinct key amino acid residues involved in substrate binding, particularly in the catalytic active site, where Gln367, Gln350, and His349 play crucial roles (Fig. 6). In addition, mutants that disrupt substrate binding, such as Q367 A (unable to bind substrate), and Q350G (unable to carry out nucleophilic attack), and H349 V (unable to provide proton) completely lose enzyme activity (Fig. 7). This suggests that, in Amuc_1547, there is a cluster of novel catalytic residues, with Gln367 involved in substrate binding and stabilization, providing the necessary spatial orientation for the catalytic reaction. Additionally, Gln350 executes nucleophilic attack via its side-chain amino group, while His349 may play a dual role: stabilizing the catalytic residue Gln350 and assisting in the protonation of the transition state (Supplementary Fig. 8). These findings redefine the catalytic paradigm of sialidases and highlight *A. muciniphila* evolutionary innovation in adapting to gut mucin degradation, positioning Amuc_1547 as a key mediator of host-microbiota symbiosis.

*A. muciniphila* encodes four sialidases (Amuc_0623, Amuc_0625, Amuc_1835, Amuc_1547), which all share a six-bladed propeller folding pattern and possess a relatively conserved S-x-D-x-G-x-x-W motif (Supplementary Fig. 2&3). However, they exhibit relatively low conservation in sequence homology and key catalytic residues for substrate catalysis (Figs. 5, 6, 7, Supplementary Fig. 3). Notably, the metal ion-binding pocket, featuring Glu289, Glu299, and Asp300 unique to Amuc_1547, is not conserved among the other three sialidases in *A. muciniphila* (Fig. 2, Supplementary Fig. 3). This suggests that the four sialidases of *A. muciniphila* may have different stabilities and catalytic activities in the gut environment. These results also indirectly confirm the reported differences in pH range adaptation, divalent metal ion influence, and hydrolysis rates on different substrates by the four sialidases in *A. muciniphila * [13].

Although this study reveals some unique structural and substrate catalytic features of Amuc_1547, supporting its potential role in host health, there are still several limitations. First, the precise structural dynamics during catalysis, such as real-time substrate interactions, need further exploration using advanced techniques like time-resolved X-ray crystallography or cryo-EM. Second, while we focused on in vitro enzyme activity, the broader physiological relevance of Amuc_1547 regulation by metal ions and carbohydrates in the gut microbiome and host immune response remains unclear. Future studies should investigate how environmental factors and diet influence in vivo function. Additionally, *A. muciniphila* encodes multiple sialidases, and a more comprehensive comparison of these enzymes could reveal their functional diversity and roles in gut health regulation. Overall, more research is needed to fully understand the biological functions of sialidases in *A. muciniphila* and their potential therapeutic implications.

## Conclusion

This study resolves the magnesium-bound crystal structure of *A. muciniphila* sialidase Amuc_1547, revealing a six-bladed β-propeller catalytic domain with a conserved S-x-D-x-G-x-x-W motif and a unique metal-binding pocket, alongside a CBM-like β-sandwich domain containing a putative carbohydrate-binding site. Functional assays demonstrate that magnesium ions and glycans enhance enzymatic activity. Structural and mechanistic analyses establish that Amuc_1547 diverges from canonical GH33 sialidases by employing a non-classical catalytic triad (Gln367, Gln350, His349) instead of the conserved arginine triplet and nucleophilic Tyr/Glu pairs. Molecular docking further identifies residues critical for binding SIA and 6′SL, while mutagenesis of key residues in the catalytic and substrate-binding regions significantly impair hydrolysis. These findings define Amuc_1547 as a mechanistic archetype of the GH181 family, providing structural and functional insights into how *A. muciniphila* sialidases drive mucin degradation to regulate gut microbial ecology and host interactions.

## Materials and methods

### Bacterial growth conditions and gene cloning

*A. muciniphila* (ATCC BAA- 835) was grown anaerobically at 37℃ in BHI media with 0.05% hog gastric mucin III (Sigma Aldrich). Growth was monitored by OD600 measurements (NanoDrop 2000, Thermo Fisher). The Amuc_1547 coding region (residues Q19-K595, UniProt: B2ULI1), excluding the signal peptide, was amplified via PCR using the Gene Touch machine (BIOER, HangZhou, China). The gene was cloned into pET- 22b with a 6His-tag at the 3'end using ClonExpress II (Vazyme), yielding pET22b-Amuc_1547 - 6His.

### Protein expression and purification

Am_1547 was heterologously expressed in *E. coli* BL21(DE3) at 18℃ using 0.4 mM IPTG induction during mid-log phase. Cell pellets obtained by centrifugation were lysed via sonication in Tris-buffered saline (25 mM Tris–HCl pH 7.5, 150 mM NaCl). The clarified lysate underwent Ni–NTA affinity purification (Sigma-Aldrich) with imidazole gradient elution (30 to 300 mM). Target protein was buffer-exchanged through size-exclusion chromatography (Superdex 200 10/300 GL, GE Healthcare) in stabilization buffer (25 mM Tris–HCl pH 7.5, 150 mM NaCl, 5% glycerol) and cryopreserved at − 80℃.

### Crystallization

The protein complex (10 mg/mL) was screened using commercial crystallization suites (Hampton Research/Rigaku) via high-throughput sitting-drop vapor diffusion at 16 °C. Promising hits from Index HT and PEG-based screens were optimized via sitting-drop vapor diffusion as previously described [24]. The final X-ray suitable crystals were obtained from the condition of 0.2 M MgCl_2_·6H_2_O, 0.1 M Bis–Tris, pH 6.5, 25% W/V PEG3350.

### Data collection and Structure determination, refinement

SeMet-derivatized crystals were cryoprotected and subjected to X-ray diffraction at Shanghai Synchrotron Radiation Facility (SSRF, Shanghai, China). Single-wavelength anomalous dispersion (SAD) data were processed through an automated pipeline: The selenomethionine sites were determined using shelx, initial phases were resolved by CCP4, followed by iterative model rebuilding in Coot and refinement using PHENIX.Refine. PyMOL was used to prepare molecular graphic images. The PDB code of Amuc_1547 was 8HLS.

### Measurements of enzymatic activity

All mutant proteins were obtained using the same protease purification method as the wild-type protein, with purities maintained above 90%. All metal ion reagents were purchased from Beyotime, Shanghai. NAD + was purchased from Yuanye Bio-Technology, Shanghai (Cat. V33581), α-Lactose (MCE, Cat. HY-N2514), α-D-glucopyranosyl phenylacetic (Yuanye Bio-Technology, Cat. S44732 - 1 g), D-Lactose (Yuanye Bio-Technology, Cat. S74660 - 100 g), and D-Glucose (Yuanye Bio-Technology, Cat. T90667 - 1 ml).

For the measurement of mutant kinetic parameters, the purified enzymes (400 nM) were incubated with different concentrations of 4MU-Neu5 Ac (6.25 μM, 12.5 μM, 25 μM, 50 μM, 100 μM, 200 μM, 400 μM, 600 μM) in buffer 25 mM Tris, 150 mM NaCl, pH 7.5, at 37℃ [25]. For testing the effect of ions and carbohydrates (NAD + and oligosaccharides) on enzyme activity, the purified enzymes (400 nM) were incubated with 100 mM 4MU-Neu5 Ac (Sigma, Cat. M8639) in enzyme-free water at 37 °C. The final concentration of all ions and carbohydrates (NAD + and oligosaccharides) was 2 mM (with Alpha-D-glucopyranose pentabenzoate having poor water solubility, which may account for not meeting the concentration condition).

The total reaction volume was 100 μL in a black opaque 96-well plate. The progression of the action was monitored by the release of 4MU using the fluorescence and absorbance spectrophotometer (TECAN Spark® Multi-Mode Microplate Reader) with an excitation at 373 nm and an emission at 448 nm. The kinetic parameters, averaged from three independent experiments, were calculated via nonlinear regression analysis (Prism 8, GraphPad) fitting raw data to the Michaelis–Menten equation, with error bars representing s.e.m.

### System preparation and molecular docking

The Amuc_1547 crystal structure was computationally processed using molecular modeling protocols to remove non-structural solvent/ion, followed by optimization of the protonation states under neutral pH conditions. The Sialic acid (SIA) and 6'-Sialyllactose (6'SL) were docked with 200 independent runs into the active site of Amuc_1547 using Autodock4.2.6. The grid box was 60 A° × 60 A° × 60 A° with a grid spacing of 0.375 A°. Lamarckian Genetic Algorithm [LGA] was applied to docking simulations. This process began with an initial population of 150 individuals and allowed for up to 2,500,000 energy evaluations per docking run. All other parameters were maintained at their default values.

For the structures and interactions of Amuc_1547 enzyme with its substrates SIA and 6'SL prediction, we utilized AlphaFold 3 (AF3), a powerful computational tool for high-accuracy biomolecular structure prediction [26]. The predicted structures were analyzed with molecular visualization tools like PyMOL to examine binding sites and key interactions. Their confidence was assessed using pLDDT scores.

### System preparation and molecular dynamics

To elucidate the interaction mechanisms between Amuc_1547 enzyme and its substrates SIA and 6'SL, molecular dynamics (MD) simulations were performed using the GROMACS software package [27]. The enzyme structure was solvated in a truncated octahedron box with TIP3P water molecules, ensuring a minimum distance of 1.0 nm between the solute and the box edge. NaCl ions were added to neutralize the system charge and maintain physiological ionic strength. Molecular dynamics simulations were performed as follows: steepest descent energy minimization, followed by NVT heating (0 K to 300 K over 100 ps; velocity rescaling, coupling constant 0.1 ps) and NPT equilibration (300 K, 1 atm pressure; Parrinello-Rahman barostat, coupling constant 2 ps) [28]. The production MD simulation was carried out for 50 ns with a time step of 2 fs, employing the Particle Mesh Ewald (PME) method for long-range electrostatic interactions and a cutoff radius of 1.0 nm for van der Waals and Coulomb interactions. The AMBER99SB-ILDN force field was used for the protein, and the TIP3P model was employed for water. Simulation data were analyzed to evaluate substrate binding stability, interaction energies between key amino acid residues and substrates, and conformational changes in the enzyme. The reliability of the simulation results was validated by comparison with experimental data.

## Supplementary Information

Supplementary Material 1

## Data Availability

All data generated or analyzed during this study are included in this article and additional data are available from the corresponding author upon reasonable request.
