# Supplementary material for: Structural and functional insights into metal coordination and substrate recognition of Akkermansia muciniphila sialidase Amuc_1547
Source: Mol Biomed. 2025 Apr 23;6:24. doi: 10.1186/s43556-025-00265-8 (PMC12018670; doi:10.1186/s43556-025-00265-8)
Supplement: Supplementary file 1 — Supplementary Material 1 [file 43556_2025_265_MOESM1_ESM.docx]

**Structural and Functional Insights into Metal Coordination and Substrate Recognition of *Akkermansia muciniphila* Sialidase Amuc_1547**

Tao Li^1#^, Xin-yue Tang^2#^, Yi-bo Zhu^3#^, Ning-lin Zhao^2^, Ying-jie Song^4^, Lihui He^5^, Xing-yu Mou^2^, Chunlei Ge^1^, Zhenpu Chen^1^，Hai Zhang^1^, Xiaoxuan Yao^1^, Xiaoyuan Hu^1^，Jiaxing Cheng^1^，Hong Yao^1^, Rui Bao^2*^.

^1^Cancer Biotherapy Center & Cancer Research Institute, Yunnan Cancer Hospital, The Third Affiliated Hospital of Kunming Medical University, Peking University Cancer Hospital Yunnan, Kunming, China.

^2^Division of Infectious Diseases, State Key Laboratory of Biotherapy and Center of Infectious Diseases, West China Hospital, Sichuan University, Chengdu, China.

^3^Accurate Biotechnology (Hunan) Co., Ltd, Changsha 410006, China.

^4^College of Life Science, Sichuan Normal University, Chengdu 610101, China

^5^Department of Pharmacy, Chengdu Second People*'*s Hospital, Chengdu, China.

* **Corresponding Author:** Rui Bao: [baorui@scu.edu.cn](mailto:baorui@scu.edu.cn)

^#^Authors contributed equally to this work.

Supplementary Material

Supplementary Table 1 Data collection and model refinement statistics.

| **Data collection** | 8HLS |
| --- | --- |
| X-ray source | BL17U |
| Wavelength(A$^{\circ}$) | 0.979 |
| Space group | P12_1_1 |
| Unit cell parameters | A=72.16 b=56.771 c=145.932 |
| Resolution range (A$^{\circ}$) | 21.64-2.042 (2.115-2.042) |
| Total/unique reflections | 73,041 /6,090 |
| Completeness (%) | 97.01 (81.88) |
| Mean I/sigma I | 30.08 |
| **Model refinement** | Phenix refine 1.19.2 |
| Rwork/Rfree (%) | 17.97/22.83 |
| Rmsd in bond lengths (A$^{\circ}$)/angles (◦) | 0.018/1.54 |
| **Ramachandran plot** |  |
| Favored (%) | 96.32 |
| Allowed (%) | 3.68 |
| Outliers (%) | 0 |


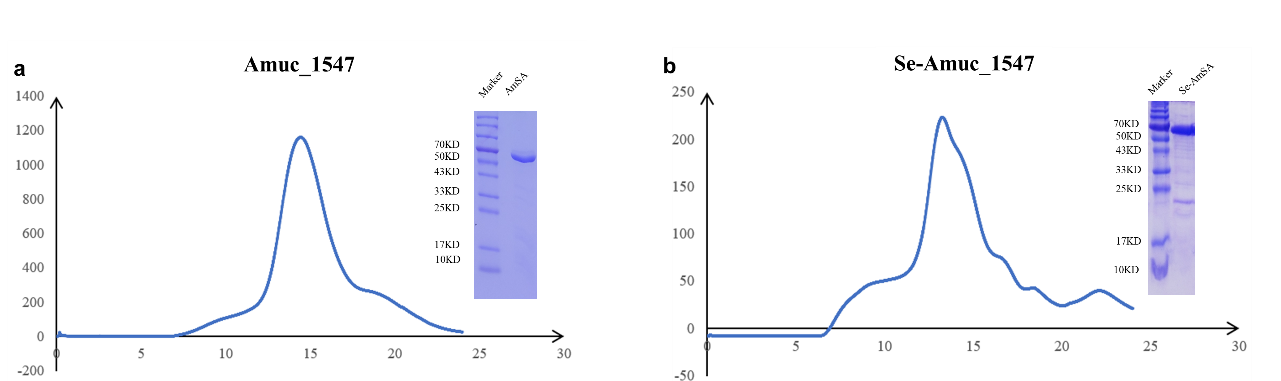


Supplementary Figure 1. Protein purification results of Amuc_1547 and Se-Amuc_1547. A. Molecular exclusion chromatography and SDS-PAGE result of Amuc_1547. B. Molecular exclusion chromatography and SDS-PAGE electrophoresis result of Se- Amuc_1547.


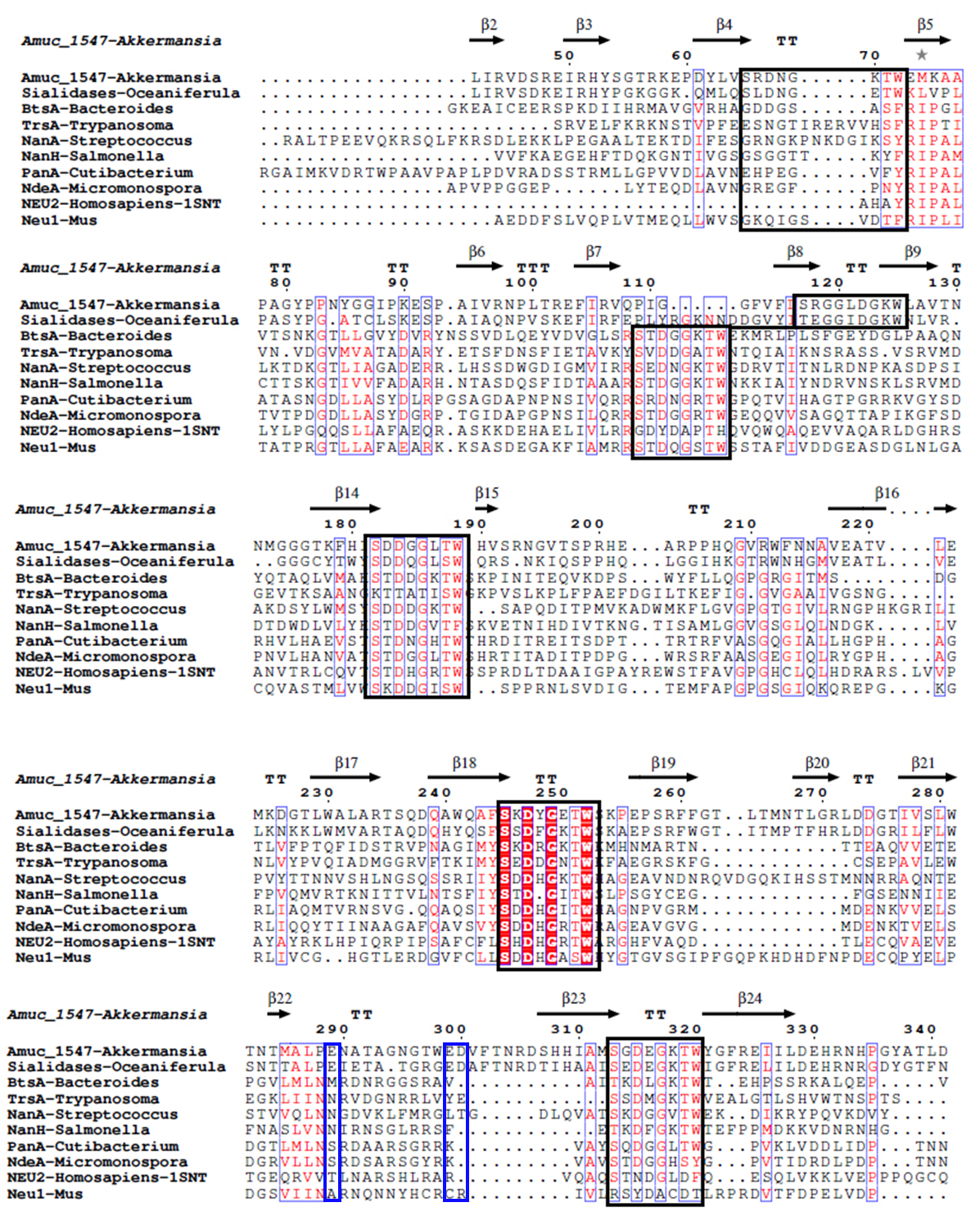


Supplementary Figure 2. Amino acid sequence alignment of Amuc_1547 with representative sialidases members of the GH33 family with a 6-bladed propeller fold. Sialidases from *Oceaniferula marina* (PDB code: 4X47), BtsA from *Bacteroides thetaiotaomicron* (PDB code: 4BBW), TrsA from *Trypanosoma rangeli* (PDB code: 1MZ5), NanA from *Streptococcus pneumoniae* (PDB code: 5KKY), NanH from *Salmonella enterica* (PDB code: 1DIL), PanA from *Cutibacterium acnes* (PDB code: 7LBU), NdeA from *Micromonospora viridifaciens* (PDB code: 1EUR), NEU2 from Homo sapiens (PDB code: 1SNT), and Neu1 from Mus musculus (PDB code: 8DU5). S-x-D-x-G-x-x-W motifs within the 6-fold β-propeller catalytic domain is marked with a black box, and key amino acid sites in the metal ion binding pocket are marked with a blue box. The sequence alignment results were generated by ESPript3 (https://espript.ibcp.fr/ESPript/ESPript/).


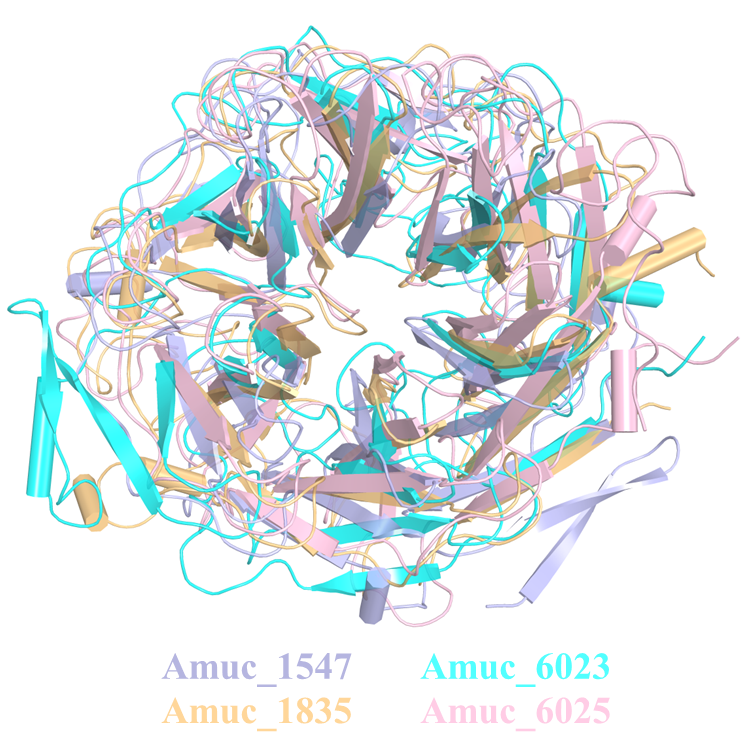


Supplementary Figure 3. Structural comparison of Amuc_1547 with three other sialidases (Amuc_0623, Amuc_0625 and Amuc_1835) from *A. muciniphila*. The structures of Amuc_0623, Amuc_0625 and Amuc_1835 were predicted by AlphaFold3. All four sialidases from *A. muciniphila*, possess a catalytic domain with a 6-blade propeller folding pattern.


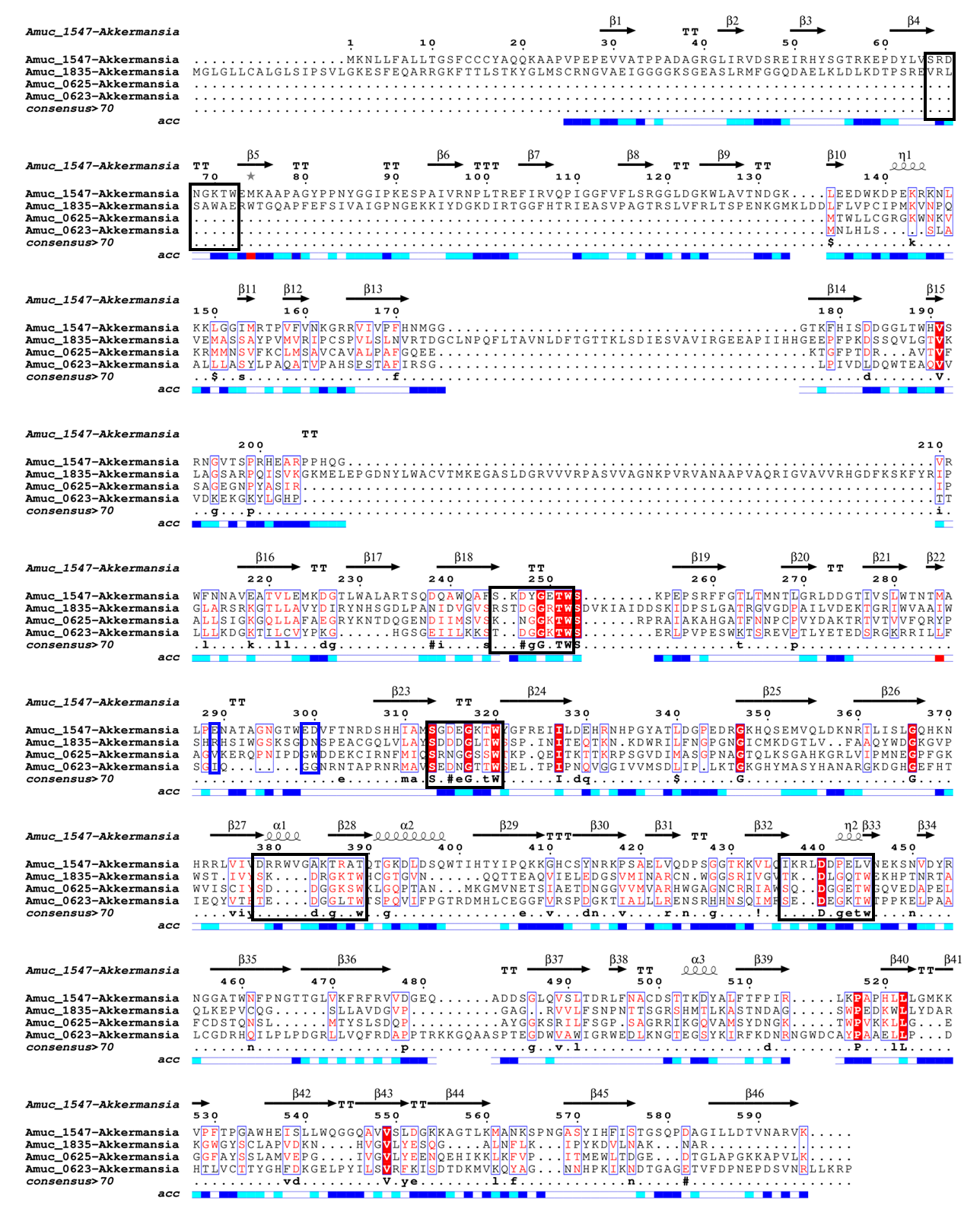


Supplementary Figure 4. Amino acid sequence alignment of Amuc_1547 with three other sialidases (Amuc_0623, Amuc_0625 and Amuc_1835) from *A. muciniphila*. S-x-D-x-G-x-x-W motifs within the 6-fold β-propeller catalytic domain is marked with a black box, and key amino acid sites in the metal ion binding pocket are marked with a blue box. The sequence alignment results were generated by ESPript3 (https://espript.ibcp.fr/ESPript/ESPript/).


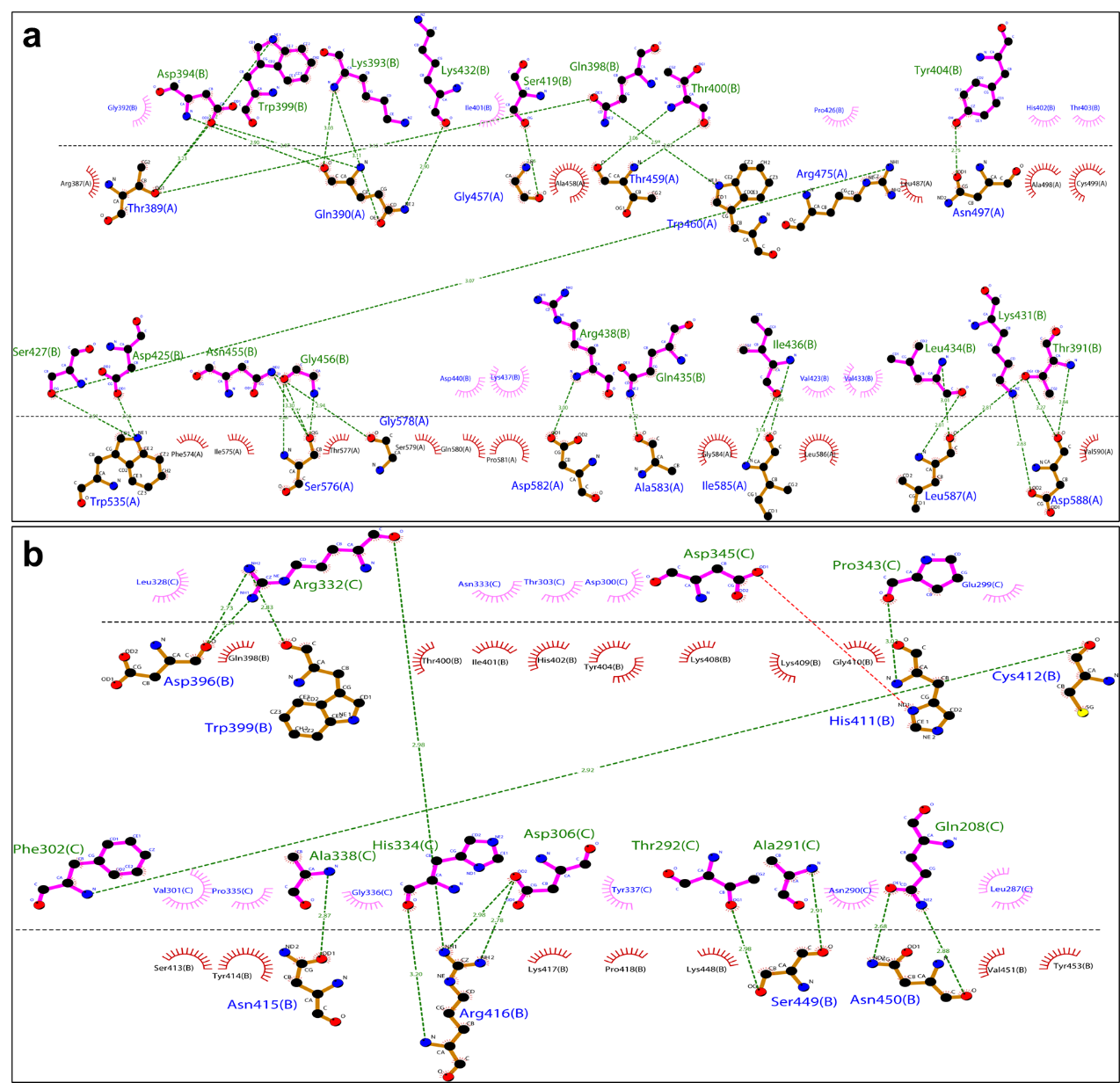


Supplementary Figure 5. Interface interactions of the linker domain with catalytic domain and CBM-like domains in Amuc_1547. The interaction pattern was generated by cLigplus software. (a). The interaction pattern between linker domain and catalytic domain. (b). The interaction pattern between Linker Domain and β-Sandwich CBM-like Domain.

**
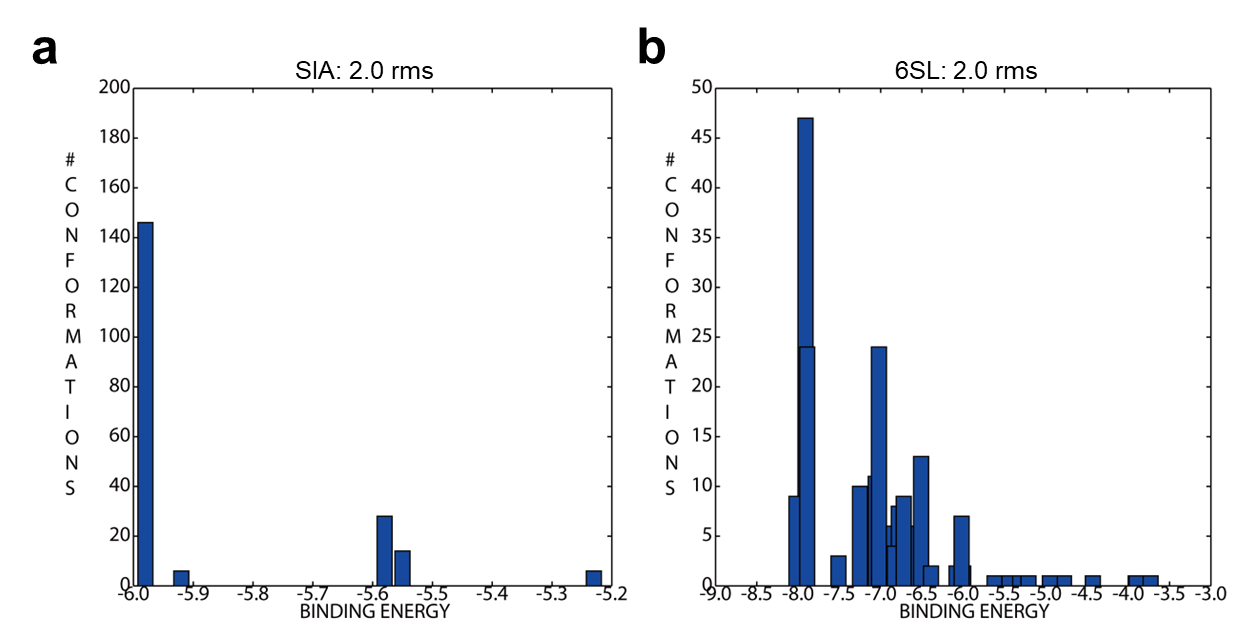
**

Supplementary Figure 6. Statistical analysis of docking poses was performed based on Cα root-mean-square deviation (RMSD) values under 2 Å. A total of 200 docking poses were classified into different clusters, with the majority of poses being in the lowest energy cluster.


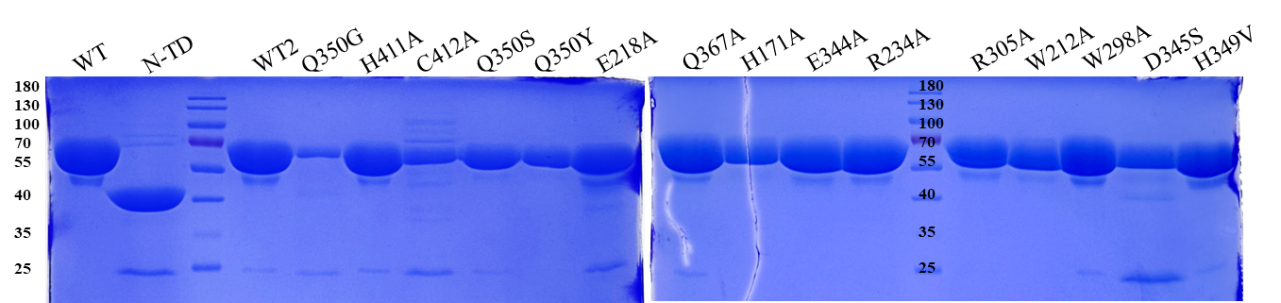


Supplementary Figure 7. SDS-PAGE analysis was used to determine the molecular weight and purity of Amuc_1547 and its mutants. All proteins were obtained after sequential purification by nickel affinity chromatography and size-exclusion chromatography. The molecular weight of the Amuc_1547-NTD variant is 41 kDa, while the other variants are all 64 kDa.

Supplementary Table 2. The expression levels for Amuc_1547 and its mutants, showing the expression levels per liter of bacterial culture.

| **Function** | **Mutants** | **Concentration**  **(mg/ml)** | **Volume**  **(ml)** | **Expression Level**  **(L^-1^)** |
| --- | --- | --- | --- | --- |
|  | WT | 9.6 | 1.5 | 14.4mg |
|  | NTD (1-389) | 2.76 | 1 | 2.76mg |
|  | CBM-Like1(390-595) | - | - | no express |
|  | CBM-Like2(465-595) | - | - | no express |
| **Catalytic active sites** | D345S | 1.78 | 0.8 | 1.42mg |
|  | E218A | 2.1 | 1.5 | 3.15mg |
| **Nucleophilic residue sites** | H349V | 2.1 | 0.5 | 1.05mg |
|  | Q350G | 1 | 0.5 | 0.5mg |
|  | Q350S | 2 | 0.8 | 1.6mg |
|  | Q350Y | 2 | 0.9 | 1.8mg |
| **Substrate Binding** | H171A | 3.1 | 1.5 | 4.65mg |
|  | W212A | 3.2 | 1.5 | 4.8mg |
|  | W298A | 6.5 | 1.4 | 9.1mg |
|  | R234A | 5.6 | 1.5 | 8.4mg |
|  | R305A | 10 | 1 | 10mg |
|  | Q367A | 8.6 | 0.8 | 6.88mg |
|  | E344A | 4.5 | 1 | 4.5mg |
|  | H411A | 4.3 | 3 | 12.9mg |
|  | C412A | 1 | 0.8 | 0.8mg |


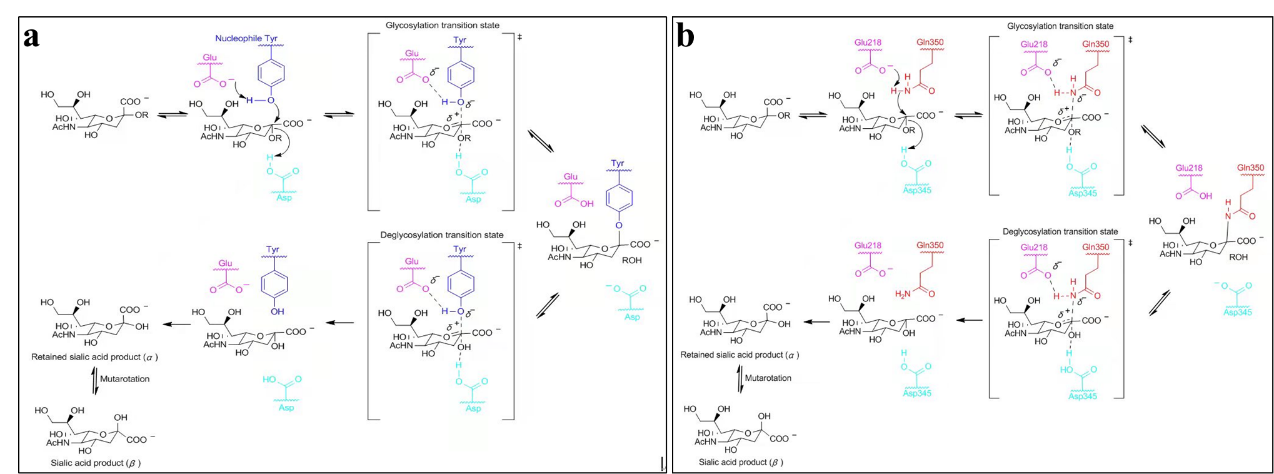


Supplementary Figure 8. The catalytic mechanism for Amuc_1547 that significantly differs from the classic sialidase family. A. The classic sialidase substrate catalytic mechanism found in the 6-bladed propeller family involves a tyrosine residue that carries out a nucleophilic attack on the C atom of the sialic acid substrate. B. In Amuc_1547, Glu350 takes the place of the tyrosine residue to perform the nucleophilic attack. His349 stabilizes the catalytic residue Glu350 and provides a proton for the transition state.
